# Antegrade stent placement and stent-in-stent placement via endoscopic ultrasound-guided hepaticogastrostomy

**DOI:** 10.1055/a-2926-9099

**Published:** 2026-08-11

**Authors:** Hidenobu Hara, Kei Onodera, Jyunko Nemoto, Shoichi Yokobori, Moe Yamashita, Kouhei Yoshino, Shinya Sakita

**Affiliations:** 1 Department of Gastroenterology 53327 Yokohama City Minato Red Cross HospitalYokohamaKanagawaJapan

**Fig. 1:**
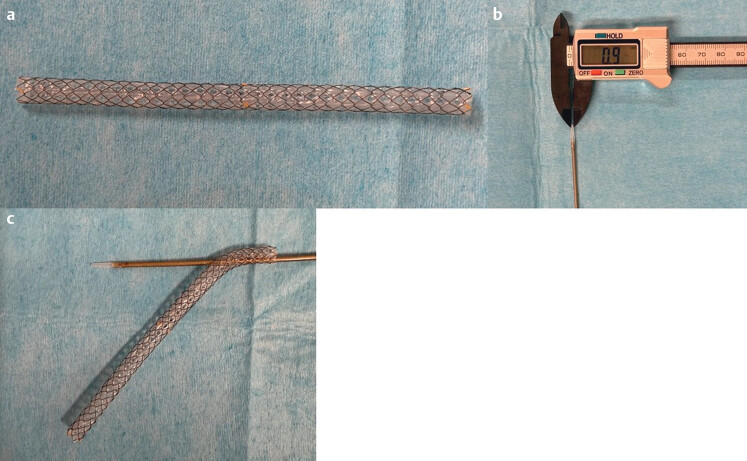
A multi-hole self-expandable metal stent with a 5.9-Fr delivery system used in the procedure. ( **a** ) The stent has multiple 1.5-mm side holes. ( **b** ) The delivery system has a diameter of 5.9 Fr and a 0.9-mm distal tip. ( **c** ) The tapered distal tip facilitates passage through the stent mesh.

Endoscopic ultrasound-guided hepaticogastrostomy can be combined with antegrade and bridging stent placement. 1​ 2​ 3​ ​4​ A 6-mm multi-hole metal stent with a 5.9-Fr delivery system and a tapered 0.9-mm tip may facilitate stent-in-stent placement through the stent mesh ( Fig. 1 ). We report double-guidewire-assisted multidirectional biliary drainage using these stents ( Video 1 ). 5​

**Video 1** Biliary drainage with antegrade stenting and stent-in-stent placement via endoscopic ultrasound-guided hepaticogastrostomy for bismuth type IIIb malignant hilar biliary obstruction in Roux-en-Y anatomy.


**Fig. 2:**
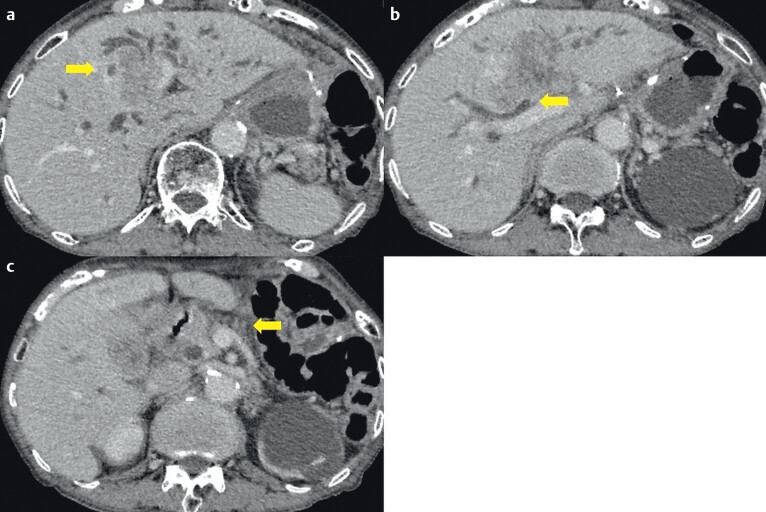
Preprocedural contrast-enhanced computed tomography. ( **a** ) An axial image showing obstruction of the B4 bile duct by the tumor (arrow). ( **b** ) An axial image showing dilatation of the right anterior and posterior sectoral ducts and separation of the right and left hepatic ducts (arrow). ( **c** ) A peritoneal nodule suggestive of dissemination is visible (arrow).

An 85-year-old man with previous distal gastrectomy and Roux-en-Y reconstruction for gastric cancer developed acute cholangitis due to bismuth type IIIb malignant hilar biliary obstruction from liver metastases invading the hepatic hilum ( Fig. 2a and b ). Given the altered anatomy and peritoneal dissemination ( Fig. 2c ), endoscopic ultrasound-guided biliary drainage was selected.

**Fig. 3:**
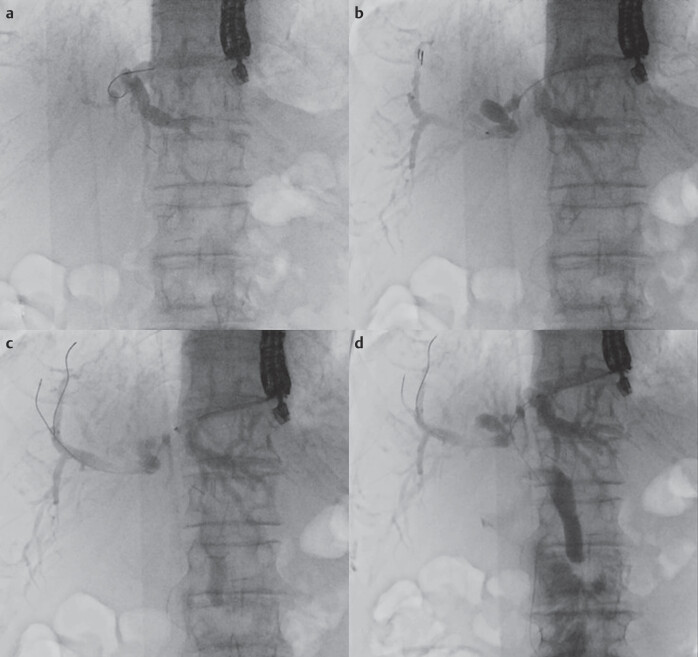
Fluoroscopic images during guidewire placement. ( **a** ) Cholangiography demonstrates the separation of the right and left hepatic ducts. ( **b** ) Contrast injection from the right anterior sectoral duct opacifies the posterior sectoral duct. ( **c** ) An additional 0.035-inch guidewire reduces the acute angulation of the access route. ( **d** ) A 0.025-inch guidewire is advanced across the hilar obstruction and through the major papilla into the duodenum.

**Fig. 4:**
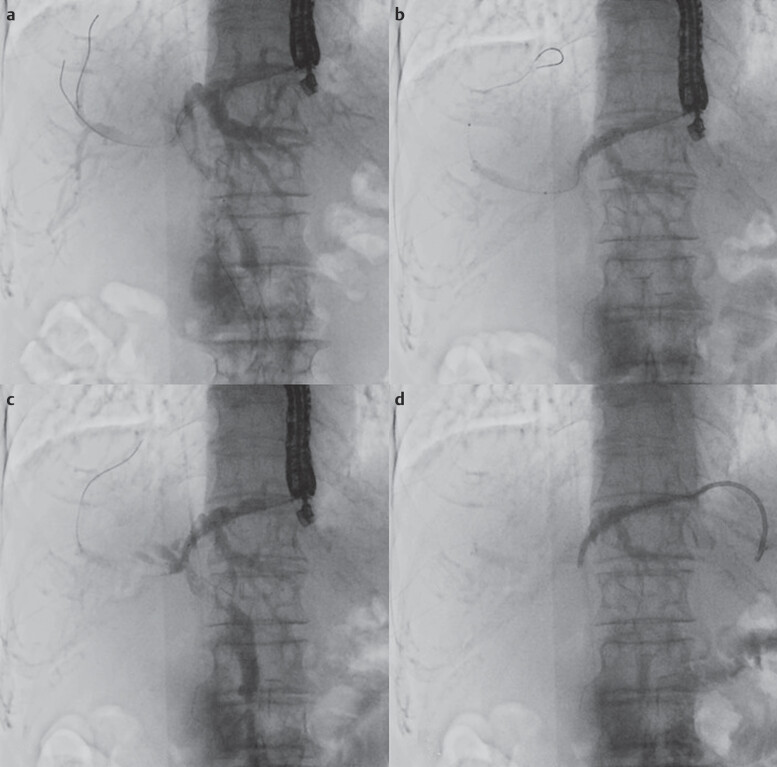
Fluoroscopic images of endoscopic ultrasound-guided hepaticogastrostomy with antegrade stenting and stent-in-stent placement. ( **a** ) A 6-mm×12-cm multi-hole metal stent is deployed antegradely from the duodenum to the left hepatic duct. ( **b** ) After traversal of the first stent mesh, stent-in-stent placement is performed using a 6-mm×10-cm multi-hole metal stent deployed from the right anterior sectoral duct to the left hepatic duct. ( **c** ) Final cholangiography confirmed the drainage of the left hepatic duct, both right sectoral ducts, and the distal bile duct across the papilla. ( **d** ) A 7-Fr, 15-cm plastic stent is placed across the hepaticogastrostomy tract.

The B2 bile duct was punctured with a 19-gauge needle, and a 0.025-inch guidewire was advanced into the right hepatic duct through a double-lumen catheter. Cholangiography showed the separation of the right and left hepatic ducts and opacification of the posterior sectoral ducts ( Fig. 3a and b ). Because severe angulation impeded catheter advancement, an additional 0.035-inch guidewire was placed to reduce the angulation and support catheter advancement ( Fig. 3c ). A second 0.025-inch guidewire was advanced across the hilar strictures into the duodenum ( Fig. 3d ). A 6-mm×12-cm multi-hole metal stent was deployed antegradely from the duodenum to the left hepatic duct ( Fig. 4a ). Guided by the retained right-sided wire, the stent mesh was traversed, and a 0.025-inch guidewire was advanced into the right anterior sectoral duct. A 6-mm×10-cm multi-hole stent was then deployed to the left hepatic duct using a stent-in-stent bridging technique ( Fig. 4b ). Final cholangiography confirmed adequate biliary drainage ( Fig. 4c ). A 7-Fr plastic stent was placed across the hepaticogastrostomy tract ( Fig. 4d ). The patient became afebrile the next day, had no adverse events, and was discharged on postprocedural day 9.

This strategy enabled bilateral biliary drainage with transpapillary stenting in malignant hilar biliary obstruction with surgically altered anatomy.

Endoscopy_UCTN_Code_TTT_1AS_2AH
